# Supplementary material for: B-cell activating factor (BAFF) and its receptors’ expression in pediatric nephrotic syndrome is associated with worse prognosis
Source: PLoS One. 2022 Nov 18;17(11):e0277800. doi: 10.1371/journal.pone.0277800 (PMC9674176; doi:10.1371/journal.pone.0277800)
Supplement: Attachment — Submitted filename: Reviewer letter NS 02aug2022.docx [file pone.0277800.s001.docx]

Aug 04, 2022

**PLOS ONE**

**Re: BAFF and its receptors’ expression in pediatric nephrotic syndrome is associated with a worse prognosis**

Dear Editor

Thank you for reviewing our manuscript and for the reviewers’ comments. We have addressed the concerns. Please find our comments and responses to the concerns below. For added clarity about the point-to-point reply, we have first repeated the requests in normal font, added our comments in italics, and changes to the manuscript were indicated in highlighted text. We hope that the edited manuscript is now acceptable for publication and wish to resubmit it.

**Reviewer 1**

1 .Please reference paragraph 2 in Introduction section (lines 93-96).

***Answer:*** *We added some references to the paragraph.*

“The most common causes of NS in pediatric patients are minimal change disease (MCD) in up to 90% of cases (associated with good prognosis) [1] and focal segmental glomerulosclerosis (FSGS) in 10-15% of cases [2]. The latter is associated with a worse prognosis and risk of progression to chronic kidney disease (CKD) [3].

2. Was there a reason not to comment on cyclophosphamide as a treatment for NS? (lines 106-108). I recommend that all immunosuppressive agents are addressed adequately in order to avoid bias.

***Answer****: We thank the reviewer's comment. Most NS patients, around 80%, can be treated only with steroids. In Steroid-Resistant Nephrotic Syndrome (SRNS) patients, we add immunosuppressor agents. Of our patients, 12 were SRNS; of them, five patients received cyclophosphamide; 2 patients, cyclosporine; 1 patient, mycophenolate mofetil; 2 patients, rituximab; 1 patient, tacrolimus and 1 patient cyclophosphamide six-months regimen and then was changed to cyclosporine due to therapeutic failure. We decided to add this last patient to the cyclosporine group. According to the reviewer's comment, we divided SRSN patients into two groups: cyclophosphamide treated patients (n=5) and other immunosuppressors treated patients (n=7). The median eGFR in cyclophosphamide group was 83 mL/min/1.73m2 (RIC 67-120) and other immunosuppressors treated patients eGFR was 110 mL/min/1.73m2 (RIC 79-115) with no significant differences (p=0.9353). We modified table 1 and added the next paragraph below.*

**Methods section**

Among these 33 patients, the median age at inclusion was 2 years (IQR 2.0 – 5.0). The median glomerular filtration rates were 115 (104-128) mL/min/1.73 m^2^ **(Table 1).** The median time between NS diagnosis and renal biopsy was 2.1 years (IQR 0.9-3.9). Twelve (36.6%) patients were Steroid-Resistant Nephrotic Syndrome (SRNS) patients. Of them 5/12 patients received cyclophosphamide and 7/12, other immunosuppressor treatment. The median eGFR in cyclophosphamide group was 83 mL/min/1.73m2 (RIC 67-120) and other immunosuppressors group, eGFR was 110 mL/min/1.73m2 (RIC 79-115) with no significant differences (p=0.9353). The most frequent pathology diagnosed by biopsy was MCD, which was found in 21 (63.6%) patients, followed by focal segmental glomerulosclerosis, which was found in 12 (36.4%) patients. **Table 2** describes the clinical characteristics at the time of renal biopsy, and **Table 3** describes the same characteristics at the last follow-up.

| **Characteristics** | **N=33** | |
| --- | --- | --- |
|  | **n** | **%** |
| **Age at diagnosis [years]*** | 2.0 (2.0-5.0) | |
| **Sex** |  |  |
| Female | 11 | 33,3 |
| Male | 22 | 66,7 |
| **Ethnicity** |  |  |
| Mestizo | 24 | 72,7 |
| Indigenous | 1 | 3,0 |
| Black | 8 | 24,2 |
| **Siblings with NS** | 5 | 15,2 |
| **Variables at diagnosis** |  | 0,0 |
| Macroscopic hematuria | 2 | 6,1 |
| Microscopic hematuria | 8 | 24,2 |
| Arterial hypertension | 11 | 33,3 |
| GFR [ml/min/1.73 m**^2^**]* | 115 (104-128) | |
| **KDIGO classification at diagnosis** |  |  |
| 1 | 29 | 87,9 |
| 2 | 3 | 9,1 |
| 3 | 1 | 3,0 |
| **Initial treatment** |  |  |
| Corticosteroids | 30 | 90,9 |
| Mycophenolate mofetil | 1 | 3,0 |
| Tacrolimus | 1 | 3,0 |
| Rituximab | 2 | 6,1 |
| ACE inhibitor | 16 | 48,5 |
| Cyclophosphamide | 5 | 15,5 |
| Cyclosporine | 3 | 9,1 |

3. Expand on the definition of frequent relapse (lines 150-151). You have used only one reference to make a strong statement. I suggest you add more data to support this statement.

***Answer:*** *We included the following reference “Lancet. 2018 Jul 7;392(10141):61-74. doi: 10.1016/S0140-6736(18)30536-1" which defines frequent relapse as ≥2 relapses within 6 months of initial response or ≥4 in any 12-month period, similar to the definition we provided in the Methods´ section (≥ 4 relapses in any 12-month period).*

*The reference was included in the reference list.*

**“Methods**

**…frequent relapse** (≥ 4 relapses in any 12-month period) [4].

4. Further clarification and referencing are needed in the Statistical analysis section: how did you decide to do such clarification such as 0-25% as one group, etc.

a reference.

***Answer:*** *We considered that the most objective way to measure our findings regarding the glomerular and interstitial biomarkers’ expression was to extrapolate the method from an already stablished one as the one from the International Society of Nephrology/Renal Pathology Society for the lupus nephritis classification (Kidney International (2018) 93, 789–796; https://doi.org/10.1016/ j.kint.2017.11.023). This was clarified on methods with the supporting reference.*

**“Statistical analysis**

Slides were read by one expert nephropathologist blinded to clinical outcome, and samples were scored according to the percentage of cells with positive expression on podocytes and interstitial inflammatory infiltrates (III) (mononuclear cells, B or T-cells), as follows: 1) 1-25% of cells stained; 2) 26-50% of cells stained; and 3) more than 50% of cells stained (extrapolated from the International Society of Nephrology/Renal Pathology Society classification for lupus nephritis [5] .

5. Can you further define the group in which the BAFF expression was positive? What treatment were those patients initially on? Were they MCD or FSGS?

**Answer.** *We thank the reviewer comment. Unfortunately, due to the low needing of biopsy in nephrotic syndrome, our sample size is low. It does not allow us to draw precise conclusions. That is why we have decided not to add the tables in the article. However, we present the tables disaggregated by type of disease below.*

| Minimal Changes | | | | | FSGS | | | | |
| --- | --- | --- | --- | --- | --- | --- | --- | --- | --- |
| **Biomarker** | **Podocytes** | | **Interstitial inflammatory infiltrate** | | **Biomarker** | **Podocytes** | | **Interstitial inflammatory infiltrate** | |
|  | **n** | **%** | **n** | **%** |  | **n** | **%** | **n** | **%** |
| **BAFF** |  |  |  |  | **BAFF** |  |  |  |  |
| Negative | 17 | 80 | 14 | 67 | Negative | 10 | 83.3 | 7 | 58.3 |
| Positive | 4 | 19 | 7 | 33 | Positive | 2 | 16.7 | 5 | 41.7 |
| **Extent*** |  |  |  |  | **Extent*** |  |  |  |  |
| 0-25% | 4 | 100 | 5 | 71.4 | 0-25% | 2 | 100 | 3 | 60 |
| 26-50% | 0 | 0 | 1 | 14.2 | 26-50% | 0 | 0 | 1 | 10 |
| ≥51% | 0 | 0 | 1 | 14.2 | ≥51% | 0 | 0 | 1 | 10 |
| **BAFF-R** |  |  |  |  | **BAFF-R** |  |  |  |  |
| Negative | 21 | 100 | 21 | 100 | Negative | 0 | 0 | 11 | 91.7 |
| Positive | 0 | 0 | 0 | 0 | Positive | 0 | 0 | 1 | 8.3 |
| **Extent*** |  |  |  |  | **Extent*** |  |  |  |  |
| 0-25% | 0 | 0 | 0 | 0 | 0-25% | 0 | 0 | 0 | 0 |
| 26-50% | 0 | 0 | 0 | 0 | 26-50% | 0 | 0 | 1 | 100 |
| ≥51% | 0 | 0 | 0 | 0 | ≥51% | 0 | 0 | 0 | 0 |
| **TACI** | 0 | 0 | 0 | 0 | **TACI** |  |  |  |  |
| Negative | 19 | 91.5 | 19 | 91.5 | Negative | 10 | 83.3 | 10 | 83.3 |
| Positive | 2 | 9.5 | 2 | 9.5 | Positive | 2 | 16.7 | 2 | 16.7 |
| **Extent*** |  |  |  |  | **Extent*** |  |  |  |  |
| 0-25% | 2 | 100 | 2 | 100 | 0-25% | 2 | 100 | 1 | 50 |
| 26-50% | 0 | 0 | 0 | 0 | 26-50% | 0 | 0 | 0 | 0 |
| ≥51% | 0 | 0 | 0 | 0 | ≥51% | 0 | 0 | 1 | 50 |
| **BCMA** |  |  |  |  | **BCMA** |  |  |  |  |
| Negative | 18 | 85.7 | 17 | 81 | Negative | 10 | 83.3 | 9 | 75 |
| Positive | 3 | 14.3 | 4 | 19 | Positive | 2 | 16.7 | 3 | 25 |
| **Extent*** |  |  |  |  | **Extent*** |  |  |  |  |
| 0-25% | 3 | 100 | 4 | 100 | 0-25% | 10 | 83.4 | 2 | 66.7 |
| 26-50% | 0 | 0 | 0 | 0 | 26-50% | 1 | 8.3 | 1 | 33.3 |
| ≥51% | 0 | 0 | 0 | 0 | ≥51% | 1 | 8.3 | 0 | 0 |

6. Based on your findings, can you provide recommendations (ie should all kidney biopsies be stained for BAFF expression? What, if any, approved for use in pediatrics drugs would you advise in those patients?)

***Answer****: Nephrotic syndrome is a disease in which immunological mechanisms are being studied. In case that BAFF and its receptors become stablished as routine biomarkers, they would be helpful in the follow-up of patients with perhaps, worst prognosis. Additionally, with the approval of anti-BAFF drugs for children, a possibility of treatment can be purposed. To make this clear in the manuscript, a reference with the FDA approval for Belimumab in children was added in the third paragraph of the discussion and a final paragraph was included to state possible recommendations from our point of view based on our findings.*

“**Discussion** (third paragraph)

The value of this study lies in it being the first description of this marker in kidney samples of patients with NS and of the long-term reduction in kidney function in patients expressing the marker. Even more interestingly, targeting BAFF therapies (i.e., belimumab) are available and approved by the Food and Drug Administration for autoimmune disorders characterized by BAFF overproduction, mainly SLE [6] and recently in patients with lupus nephritis [16] as has been demonstrated by methods of immunohistochemistry[14] and RNAseq [17] of renal biopsies, suggesting that giving anti-BAFF drugs may be a future strategy to treat patients with NS. Furthermore, belimumab has shown effectiveness on reducing anti-phospholipase A2 receptor autoantibodies and proteinuria in in primary membranous nephropathy [18].”

**“Discussion** (last paragraph)

In this way, NS is a disease in which immunological factors as BAFF and it receptors are being described, so an interest to evaluate them in the clinical practice should arise as they could imply a worst prognosis or the need for a closer follow-up in these patients. Furthermore, the availability of anti-BAFF therapies approved for pediatric patients state a possibility of treatment for patients who present those biomarkers. Prospective studies are required to confirm this hypothesis.”

**Reviewer 2**

The authors investigated the expression of BAFF and related receptors (BAFF-R, BCMA, and TACI) in pediatric nephrotic syndrome, and concluded that BAFF expression in the kidney was associated with less favorable outcomes. My comments and concerns are as follows:

The scientific rationale and hypothesis of this study are not strong and are not clearly explained in the Introduction.

***Answer:*** *To state this clearer, we modified the last paragraph of the Introduction as follows:*

“**Introduction** (last paragraph)

With the aim to provide further knowledge on any possible immunopathogenic mechanism involved in NS and as the role of BAFF and its receptors in pediatric patients with NS has not been evaluated, we investigated the expression of BAFF and its three receptors in kidney biopsies from patients with NS ~~and~~ to correlate their expression with clinical outcomes. The identification of BAFF and its receptors in renal biopsies from NS could lead to their application in the clinical practice as potential biomarkers and as a possible target of immunomodulatory therapies.”

2. The sample size is relatively small, and the patient characteristics are heterogeneous.

***Answer****: We thank the reviewer's comments. Unfortunately, given the low frequency of the need for biopsy in patients with nephrotic syndrome, our sample is limited. This means that the sample size is not probabilistic. We add this observation in the study limitation section.*

**STUDY LIMITATIONS:**

Given the nature of our study, access to medical information was restricted to the available medical charts in our center. Because the renal biopsy needing in patients with nephrotic syndrome is low (the management can be done without the biopsy in most cases), our sample size is small.

3. The overall results are mixed and hence no definition conclusion can be drawn.

***Answer:*** *To state our conclusions clearer, we complemented the discussion as follows:*

“**Discussion** (third paragraph)

The value of this study lies in it being the first description of this marker in kidney samples of patients with NS and of the long-term reduction in kidney function in patients expressing the marker. Even more interestingly, targeting BAFF therapies (i.e., belimumab) are available and approved by the Food and Drug Administration for autoimmune disorders characterized by BAFF overproduction, mainly SLE [15] (for adults since 2011, and children since 2019 FDA approves first treatment for pediatric patients with lupus | FDA )and recently in patients with lupus nephritis [16] as has been demonstrated by methods of immunohistochemistry[14] and RNAseq [17] of renal biopsies, suggesting that giving anti-BAFF drugs may be a future strategy to treat patients with NS. Furthermore, belimumab has shown effectiveness on reducing anti-phospholipase A2 receptor autoantibodies and proteinuria in in primary membranous nephropathy [18].”

**“Discussion** (last paragraph)

In this way, NS is a disease in which immunological factors as BAFF and it receptors are being described, so an interest to evaluate them in the clinical practice should arise as they could imply a worst prognosis or the need for a closer follow-up in these patients. Furthermore, the availability of anti-BAFF therapies approved for pediatric patients state a possibility of treatment for patients who present those biomarkers. Prospective studies are required to confirm this hypothesis.”

4. The choice of outcome (eGFR >90) is rather unorthodox; more conventional outcomes should be remission status (e.g. CR or PR or NR), development of CKD, doubling of Scr or ESKD.

**Answer:** *We appreciate the reviewer's comment. Although chronic kidney failure (eGFR <60mL/min/1.73m2 [KDIGO stage III or more]) is a relevant outcome to study, our objective in this study was to detect reduced kidney function, not chronic kidney failure. Also, fortunately only one of our patients had eGFR stage IV according to KDIGO, so it was impossible to perform an analysis using eGFR < 60mL/min/1.73m2 cut-off point.*

*The renal function according to steroid response was already compared in the manuscript. We present the paragraph below*

**Results**

***Renal outcomes according to response to corticosteroids***

A Kaplan-Meier curve was generated to analyze renal function defined as renal failure when GFR was ≤ 90 mL/min/1.73 m^2^ at 60 months of follow-up according to steroid response. No differences were found between steroid responders (78.1% of probability) and steroid resistants (72.9%) **(*p*=0.9718)** (**Figure 5**).

**Figure 5.** Probability of eGFR >90mL/min/1.73m2 at 60 months follow-up according to steroid response.

5.Table 4 should be tabulated according to the underlying renal pathology (MCD vs. FSGS).

***Answer****. We thank the reviewer comment. The requested changes have already been made according to the reviewer 1 observation*.

6. There are many typos in Figures 2&3 (e.g. absence, at 60 months; according to, etc).

***Answer****. We thank the reviewer comment.* The figures were modified as follow

**Figure 2.** Probability of eGFR >90mL/min/1.73m2 at 60 months follow-up according to BAFF expression in renal podocytes

**Figure 3.** Probability of eGFR >90mL/min/1.73m2 at 60 months follow-up according to BAFF expression in renal interstitial inflammatory infiltrate

**Figure 4.** Probability of eGFR >90mL/min/1.73m2 at 60 months follow-up according to BCMA expression in renal interstitial inflammatory infiltrate

**REFERENCES**

1.	Vivarelli M, Massella L, Ruggiero B, Emma F. Minimal change disease. Clin J Am Soc Nephrol. 2017;12: 332–345. doi:10.2215/CJN.05000516

2.	Shabaka A, Ribera AT, Fernández-Juárez G. Focal Segmental Glomerulosclerosis: State-of-the-Art and Clinical Perspective. Nephron. Nephron; 2020. pp. 413–427. doi:10.1159/000508099

3.	Maas RJ, Deegens JK, Smeets B, Moeller MJ, Wetzels JF. Minimal change disease and idiopathic FSGS: Manifestations of the same disease. Nature Reviews Nephrology. Nat Rev Nephrol; 2016. pp. 768–776. doi:10.1038/nrneph.2016.147

4.	Noone DG, Iijima K, Parekh R. Idiopathic nephrotic syndrome in children. The Lancet. Lancet; 2018. pp. 61–74. doi:10.1016/S0140-6736(18)30536-1

5.	Bajema IM, Wilhelmus S, Alpers CE, Bruijn JA, Colvin RB, Cook HT, et al. Revision of the International Society of Nephrology/Renal Pathology Society classification for lupus nephritis: clarification of definitions, and modified National Institutes of Health activity and chronicity indices. Kidney Int. 2018;93: 789–796. doi:10.1016/j.kint.2017.11.023

6.	Food and Drug Administration. FDA approves first treatment for pediatric patients with lupus. Am Acad Pediatr News. 2019 [cited 2 Aug 2022]. doi:10.31525/fda2-ucm636756.htm
